# Comparison of Expression of Growth Hormone-Releasing Hormone and Its Receptor Splice Variant 1 in Different Stages of Endometriosis

**Published:** 2013-03-06

**Authors:** Qiming Wang, Yueyue Wang, Xianhu Fu, Yong Huang

**Affiliations:** 1 Department of Obstetrics and Gynecology, Ningbo Women and Children’s Hospital, Ningbo, China; 2 Department of Pathology, The Second Hospital of Shaanxi Traditional Chinese Medicare University, Xianyang, China

**Keywords:** Growth Hormone Releasing Hormone, Splice Variant, Endometriosis

## Abstract

**Background:**

The present study aims to explore the significance of the expression of growth hormone-releasing hormone (GHRH) and its receptor splice variant 1 (GHRHSV1) in endometriosis (EM).

**Materials and Methods:**

In this research paper 80 EM patients who received treatment between March 2009 and September 2010 were selected, among which 20 were in stages I, II, III and IV respectively. 50 non-EM patients who underwent hysterectomy because of myoma during the same period comprised the control group. GHRH, GHRH-SV1 and their corresponding mRNA expression in eutopic endometrium and endometriotic tissue as well as ectopic endometrium were detected using immunohistochemical streptavidin-peroxidase (SP) and RT-PCR methods. Analysis of Variance (ANOVA) with Tukey Post Hoc test was used for data analysis and p<0.05 was considered significant.

**Results:**

GHRH, GHRH-SV1 and their corresponding mRNA were expressed in eutopic endometrium and endometriotic tissue as well as ectopic endometrium. The mean optical density (OD) values of the GHRH and GHRH-SV1 expression in the experimental group were significantly higher than those in the normal group (p<0.05), and the relative intensity (RI) of GHRH mRNA and GHRH-SV1 mRNA expression in the experimental group was also significantly higher (p<0.05). The mean OD values of the GHRH and GHRHSV1 expression showed significant differences among endometriotic tissue at different stages of EM (p<0.05), and the RI of GHRH and GHRH-SV1 mRNA expression also showed significant differences (p<0.05).

**Conclusion:**

GHRH and GHRH-SV1 expression levels differ significantly at different stages of endometriosis.

## Introduction

Endometriosis (EM) is the growth of cells similar to those inside of the uterus (endometrial cells), but in a location outside of the uterus (1). It is one of the most common benign diseases among women (2). However, its pathologic mechanism still remains unclear. There are differences in gene expression between endometriotic tissue and eutopic endometrium (3, 4) and the characteristics of the EM endometrium and its growth environment play important roles in the initiation and development of EM (5, 6). Growth hormone-releasing hormone (GHRH) is a polypeptide hormone containing 42- 44 amino acids. Originally, it was thought that GHRH was the product of hypothalamic secretion, and exerted its functions in the pituitary gland to activate the synthesis and secretion of growth hormone and regulate the proliferation and differentiation of pituitary somatotropes. But recent studies show that GHRH is expressed in other tissues in addition to the pituitary, suggesting that GHRH has extensive biological effects (7).

GHRH receptors contain seven transmembrane domains. They have a relatively high degree of homology with receptors such as vasoactive intestinal peptide, pituitary adenylate cyclase activating peptide and calcitonin. Therefore, GHRH can exert its extra-pituitary functions via different receptors according to different types of cells (8). It plays its roles as an autocrine growth factor in many types of tumors. Up to now, some extra-pituitary GHRH receptors have been identified and their splice variant cDNA has been detected in tumor tissues (9-11). GHRH-SV1, a splice variant of GHRH receptors, is highly similar to the pituitary GHRH receptor and can mediate and promote mitosis. It exerts its biologic effects through its integration with GHRH. The integration activates adenylate cyclase to produce cyclic adenosine monohosphate (cAMP) which is the common second signal of the GHRH receptors (12-14).

Previous study showed that GHRH is expressed in eutopic endometrium (15).This study explores whether GHRH and GHRH-SV1 are expressed in eutopic endometrium and endometriotic tissue, and analyzes the possible differences in their expression at different clinical stages of EM.

## Materials and Methods

### Samples

In this research paper, 80 EM patients were involved in the current study, whose age ranged from 22 to 48 years (35.5 ± 2.0). They were diagnosed with EM by laparoscopy or pathology after opening surgery in Ningbo Women and Children’s Hospital between March 2009 and September 2010. Among all subjects, 20 were at stages I, II, III and IV. The specimens were taken from ectopic endometrium and endometriotic tissue during operation. The control group was comprised of 50 non-EM patients who underwent hysterectomy because of myoma during the same period, with age range of 20-49 (mean 35.0 ± 2.5) years. The age range showed no significant difference compared with the experiment group (p>0.05). All specimens were not infected. All enrolled patients had regular menstrual cycles without internal complications, such as diabetes, high blood pressure, heart disease and endocrine system disease. All patients had no other endometrial diseases such as endometrial polyps, uterus gland myopathy and the merger reproductive system malignant tumors.

They didn’t receive hormonal therapy within three months before the operation. EM staging was in accordance with the revised American Society for Reproductive Medicine (rASRM).The stage and score of each patient were performed by one chief physician and two attending physician who were involved in the operation. This study was conducted with approval from the Ethics Committee of Ningbo Women & Children’s Hospital, China. Written informed consent was obtained from all participants.

### Immunohistochemical method

Each sample was divided into two fragments. One fragment was washed with saline, fixed in 10% formaldehyde and embedded in paraffin. Serial sections were then prepared at the thickness of 4 μm. The other was put into an eppendorf tube after washing with saline which was further placed into an ice cylinder, and then sent to the lab immediately for storage at -80˚C.

The procedures were performed according to the instructions indicated in the streptavidin-peroxidase (SP) kit (Beijing Zhongshan Biotechnology Co., LTD, China). After staining, slice with the brown particles represented that the tissue contained the detected material. The sections were analyzed using the image processing system and HPIAS-1000 high-resolution image analyzing software to determine the staining intensity and distribution range. Five amplified fields (40×10) were selected randomly and the mean optical density (OD) value in each field was measured for quantitative analysis.

### RNA extraction and RT-PCR

Tissue (0.1 g) was grinded into pulp and 1ml Trizol reagent (Invertrogen, USA) was added for total RNA extraction according to the manufacturer’s instructions. The absorbance of 260/280 was measured to calculate the concentration and purity of the RNA sample. Samples with a 260/280 ratio between 1.8 and 2.0 were taken for RT-PCR detection.

The procedures were performed according to the instructions indicated in the RT-PCR kit (Beijing Zhongshan Biotechnology Co., Ltd., China). The amplification conditions for GHRH included an initial pre-denaturation at 95˚C for 10 minutes followed by 40 cycles of 94˚C for 30s, 60˚C for 30 s and 72˚C for 1 minute. Glyceraldehyde-3-phosphate dehydrogenase (GAPDH) was used as an internal control. The amplification conditions for GHRH- SV1 included an initial pre-denaturation at 95˚C for 3 minutes followed by 35 cycles of 95˚C for 30s, 58˚C for 30s and 72˚C for 2 minutes. The forward and reverse primers are listed in table 1. PCR products (5 μl) were analyzed in 1.5% agarose gel electrophoresis and results were observed under the ultraviolet projection reflector. DNA Marker (TakaRa Co., LTD, China) was used as the DNA length marker. Dot intensity scanning was performed for positive straps using the digital imaging system and GAPDH correction was carried out for relative amount analyses. The expression intensity of a target gene was determined by the ratio between the absorbance of the target gene products and that of the GAPDH products.

### Statistical analysis

Data were presented by means ± standard error (Mean ± SEM), and analyzed using the SPSS 12.0 software. Analysis of Variance (ANOVA) with Tukey Post Hoc test was carried out and p<0.05 was considered statistically significant. All data were normally distributed and had homogeneous variances.

## Results

### GHRH, GHRH-SV1 expression in the normal and EM groups

**Fig 1:**
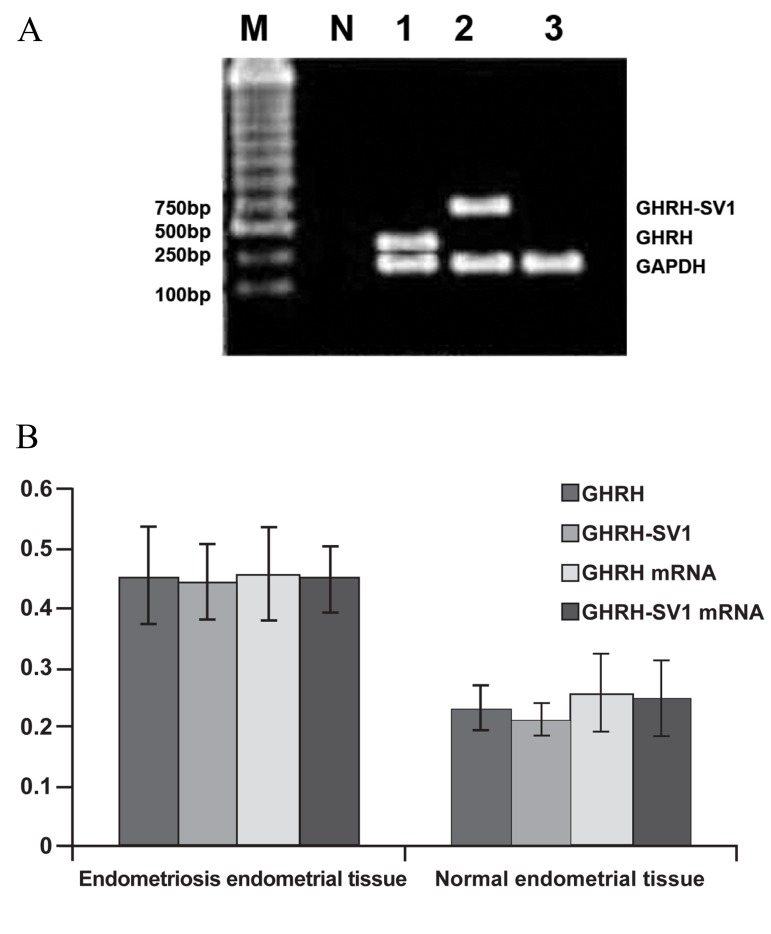
A. Electrophoresis of expression of GHRH, GHRHSV1 and the corresponding mRNA. Lane M; DNA marker, Lane N; Negative control, Lane 1; GHRH (322bp) and GAPDH (226bp), Lane 2; GHRH-SV1 (720bp) and GAPDH (226bp)and Lane 3; GAPDH (internal control 226bp). B. Analysis of expression of GHRH, GHRH-SV1 and the corresponding mRNA in normal endometrium and endometriosis tissues (Y axis represents the corresponding GHRH, GHRH-SV1 and mRNA expression level).

GHRH, GHRH-SV1 and their corresponding mRNA were expressed in eutopic endometrium and endometriotic tissue as well as ectopic endometrium. The mean OD values of GHRH and GHRHSV1 in the experimental group were significantly higher than those in the control group (p<0.05). The RI of GHRH and GHRH-SV1 mRNA in the experimental group were significantly higher than those in the control group ( p<0.05) (Table 2, Fig 1A, B). The mean OD values of GHRH and GHRH-SV1 in ectopic endometrium were significantly higher than those in endometriotic tissue (p<0.05).

**Table 1:** The primer sequence used in RT-PCR


Gene	Primer sequence (5'-3')	Length (bp)

**GHRH**	ATT TGA GCA GTG CCT CGG AG	322
	TTT GTT CTG CCC ACA TGC TG	
**GHRH-SV1**	CCT ACT GCC CTT AGG ATG CTG G ATC TCA	720
	CGG AAG TGG CAT GGC C	
**GAPDH**	GAA GGT GAA GGT CGG AGT	226
	GAA GAT GGT GAT GGG ATT TC	


**Table 2:** Expression of GHRH, GHRH-SV1 and the corresponding mRNA in eutopic endometrium and endometriotic tissue


	Endometriotic tissue	Eutopic endometrium	P (F value)

**GHRH protein**	0.4532 ± 0.0825	0.2323 ± 0.0382	<0.05 (315)
**GHRH- SV1 protein**	0.4432 ± 0.0634	0.2125 ± 0.02684	<0.05 (594)
**GHRH mRNA**	0.4576 ± 0.078	0.2573 ± 0.066	<0.05 (228)
**GHRH- SV1 mRNA**	0.4487 ± 0.056	0.2477 ± 0.065	<0.05 (350)


### GHRH, GHRH-SV1 expression in the lesion tissues at the different stages of EM

**Fig 2:**
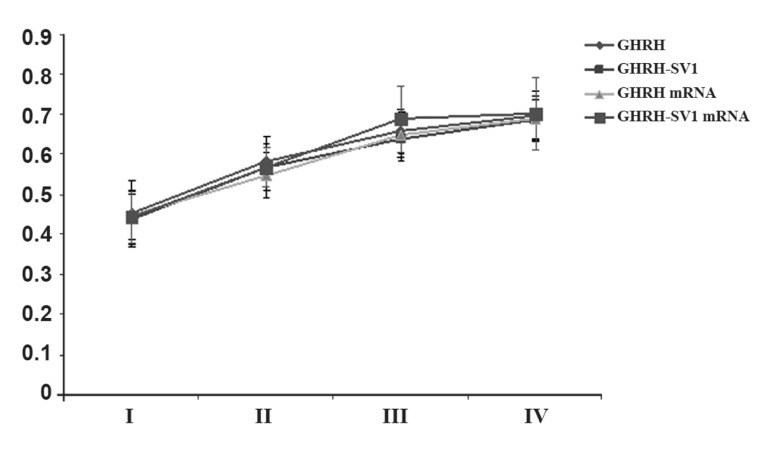
Expression of GHRH, GHRH- SV1 and the corresponding mRNA in different stages of endometriosis lesions (Y axis represents the corresponding GHRH and GHRHSV1 mRNA expression level).

As shown in table 3 and figure 2, ANOVA show significant differences in GHRH and GHRH-SV1 expression among the different stages of EM. Variance analyses show significant differences in GHRH and GHRH-SV1 mRNA expression among the different stages of EM (p<0.05).

**Table 3:** Expression of GHRH, GHRH- SV1 and the corresponding mRNA in different stages of endometriosis lesions


Item	Stage				P(F value)
	I	II	III	IV	

**GHRH protein**	0.4532 ± 0.0825	0.5832 ± 0 .0634	0.6582 ± 0.05536	0.6975 ± 0.05963	<0.05 (53)
**GHRH-SV1 protein **	0.4382 ± 0.0635	0.5682 ± 0.0574	0.6386 ± 0.05466	0.68753 ± 0.04993	<0.05 (73)
**GHRH mRNA**	0.4483 ± 0.061	0.5487 ± 0.056	0.6487 ± 0.056	0.6887 ± 0.056	<0.05 (71)
**GHRH-SV1 mRNA**	0.4432 ± 0.063	0.5686 ± 0.048	0.6887 ± 0.083	0.7021 ± 0.090	<0.05 (55)


## Discussion

Research has found that GHRH can play roles besides of the pituitary tissues and SV1 displays the closest sequence similarity to GHRH receptors among various splice variants which can express GHRH receptors in human tumors (16). It is a type of functional receptor which can induce the mitosis in tumors as well as other GHRH-related signals (17, 18). Positive-stained GHRH receptors are found in the human endometrium (19). This research prompts that GHRH and the splice variants of GHRH receptors may have important roles in the pathogenesis of EM.

Our study shows that GHRH and GHRH-SV1 are expressed in eutopic endometrium and endometriotic tissue as well as ectopic endometrium. The GHRH and SV1 expression in endometriotic tissue is significantly higher than that in eutopic endometrium. In this study, we find that the GHRH and SV1 expression in ectopic endometrium is significantly higher than that in endometriotic tissue. Different stages of EM also show significant differences in GHRH and SV1 expression. Recently, Fu et al. (20) found when ectopic endometrial stromal cells (ESC) were isolated and cultured with growth hormone-releasing hormone, the production of cAMP and the incorporation of 5-bromo-2'-deoxyuridine in SV1-expressing ESC is stimulated. These results suggest that the interaction between GHRH and SV1 may be a possible mechanism in the initiation and development of EM. In this study, the differences in the GHRH and SV1 expression among different stages of EM exhibit a non-linear relationship between the stages and GHRH and SV1 expression (Fig 2), suggesting that other signaling pathways may also play roles in the process.

Treatment of endometriosis is a difficult matter. The finding on GHRH and SV1 will represent a new approach. Annunziata et al. found that the GHRH antagonist JV-1-36 inhibited endometriotic cell proliferation and survival, suggesting that the GHRH antagonist may represent a promising tool for treatment of endometriosis (21).

## Conclusion

To sum up, the actual mechanism underlying EM still remains unclear. The current study is expected to provide a possible explanation for the pathogenesis of EM as well as an option in its treatment.
